# Liraglutide attenuates aluminum chloride-induced Alzheimer’s disease in rats by modulating the oxLDL/LPA/LPAR1 pathway

**DOI:** 10.1038/s42003-026-09531-z

**Published:** 2026-02-11

**Authors:** Nada F. Abo El-Magd, Nehal M. Ramadan, Salma M. Eraky

**Affiliations:** 1 Biochemistry Department, Faculty of Pharmacy, Mansoura University, Mansoura, Egypt; 2 Clinical Pharmacology Department, Faculty of Medicine, Mansoura University, Mansoura, Egypt

**Keywords:** Molecular neuroscience, Alzheimer's disease

## Abstract

Aluminum toxicity in rodents is well documented to be used for inducing experimental models that mimic the clinical phenotypes of Alzheimer’s disease (AD). Liraglutide is a well-known antidiabetic drug promising for modulating neurodegenerative conditions. Thus, investigating the ameliorative effects of Liraglutide on AD induced by aluminum chloride (AlCl_3_), highlighting the role of lysophosphatidic acid (LPA)/ β-secretase 1 (BACE1), is promising. Male rats are subdivided into four groups. Except for the normal group, animals are subjected to daily administration of AlCl_3_ (70 mg/kg, i.p.) for 45 days. Along with AlCl_3_, Liraglutide (0.3 mg/kg twice daily, s.c.) and Donepezil (1 mg/kg daily, i.p.) therapy are administered in AlCl3 + Lira and AlCl_3_ + Done groups, respectively. Liraglutide significantly ameliorates AlCl_3_-induced anxiety, depression-like behaviors, and deficits in memory functions. Liraglutide therapy retains the histopathological structure of the brain, with antioxidant and anti-apoptotic abilities. Moreover, Liraglutide successfully decreases hippocampal levels of oxidized low-density lipoprotein (oxLDL), LPA, lysophosphatidic acid receptor 1 (LPAR1), and β-secretase 1 (BACE1) compared with the AlCl_3_ group. Thus, liraglutide shows neuroprotective effects mediated by downregulation of the oxLDL/LPA/LPAR1/BACE1 pathway, which is studied for the first time to our knowledge.

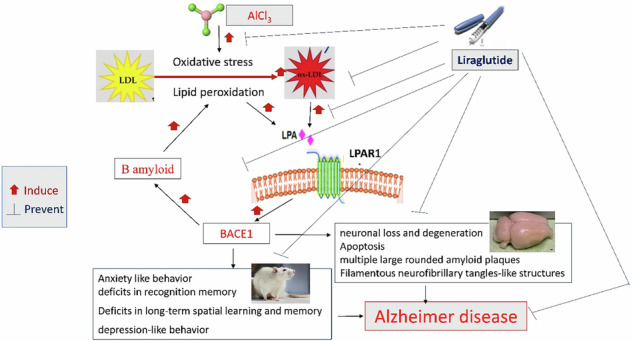

## Abstract (web summary)

Mechanistic insights into liraglutide-mediated neuroprotection reveal modulation of oxidized LDL and lysophosphatidic acid signalling in an experimental rat model of Alzheimer’s disease.

## Introduction

Alzheimer’s disease (AD) is a type of chronic neurodegenerative condition that manifests as aging-related memory loss and cognitive decline^1,2^. AD and other dementias represent the seventh most common cause of death globally. Moreover, their incidence exceeds 55 million newly diagnosed cases every year^3^. At the same time, countless patients are unaccounted for due to several causes, such as a lack of trained professionals, inaccessible resources, geographical or cultural biases, and a lack of awareness^4^. Thus, investigating the underlying molecular mechanisms of AD and finding effective preventive/treatment options are urgently needed.

The etiology of AD is multifactorial, some factors have been stated to be involved, such as head trauma, genetics, the environment, oxidative stress, and inflammation^5,6^. Aluminum toxicity is an environmental insult that has long been used to induce experimental models that mimic the human phenotype of AD. In addition to neurofilamentous changes in the cerebral cortex, hippocampus, brainstem, and spinal cord, biochemical changes, neurological signs and progressive neurodegeneration are successfully reproduced^5,7,8^. Therefore, investigating the molecular mechanisms underlying the development of aluminum chloride (AlCl_3_)-induced AD in rats is promising.

A close connection and shared pathogenesis between obesity, diabetes (especially type 2 diabetes), and AD have been reported, although the exact mechanisms are still debatable^9,10^. Aging, diabetes, and insulin resistance have been stated as major risk factors for AD^11,12^. In addition, studies have shown that AD patients have brain insulin resistance^13,14^. Thus, AD has been called “type 3 diabetes”^15^. There is no effective treatment for AD. Therefore, studying the repurposing of antidiabetic drugs for AD is promising and will have the advantage of decreasing the development cost and time to market over standard discovery.

Liraglutide, a synthetic glucagon-like peptide-1 (GLP-1), has excellent potential in treating type-2 diabetic patients and obesity. Liraglutide has a neuroprotective or neurotrophic effect as it increases the secretion and sensitivity of insulin^16^. GLP-1 is widely present in several regions of the central nervous system (CNS), including the hypothalamus, hippocampus, cortex, striatum, substantia nigra, brainstem, and subventricular zone and is thought to have a crucial function^17,18^. Previous studies have described promising impacts of GLP-1 receptor modulation in different preclinical models of neurodegenerative events, including hyperhomocysteinemia-induced AD^16^.

Donepezil, a highly selective central-acting reversible acetylcholinesterase (AChE) inhibitor, prevents the acetylcholine breakdown at synapses, thereby increasing its availability in the brain. This enhances the cholinergic transmission and alleviates the cognitive and behavioral symptoms of AD, such as memory loss and confusion. Donepezil is an FDA-approved drug for mild to severe AD^19,20^. Thus, in the current study, we used Donepezil as a reference drug to compare its attenuating ability with Liraglutide against AlCl₃-induced AD.

Reviewing the pathological features of AD has led to the selection of one promising candidate mechanism: the deposition of beta-amyloid (Aβ) plaques in the cerebral cortex, which are produced by the two consecutive cleavages of amyloid precursor protein carried out by β-secretase (BACE1) and γ-secretase^21^. BACE1 has been reported to be elevated in AD patients^22–24^ due to different mechanisms, such as oxidative stress, energy deprivation, ischemia, and hypoxia^25^. Previous publications have also reported that oxidized low-density lipoprotein (oxLDL), probably via its most bioactive component, lysophosphatidic acid (LPA)^26,27^, contributes tightly to the etiology of AD through the upregulation of BACE1 and subsequently increases Aβ production^27,28^. The relation between GLP-1 and oxLDL/BACE1/ LPA in the progression and mediation of AD is still not well studied.

Thus, this study aimed to examine the protective effect of Liraglutide compared to the well-known acetylcholinesterase inhibitor (Donepezil) against AD induced by AlCl_3_ in rats. We focused on investigating the role of the oxLDL/LPA/BACE1 molecular pathway. To the best of our knowledge, the effect of Liraglutide on LPA/LPAR1 has not been explored.

## Materials and methods

### Chemicals and drugs

The AlCl_3_ hydrate (AlCl_3_·6H2O) was acquired from El-Gomhouria Chemical Company, Egypt. Liraglutide and Donepezil hydrochloride were purchased from Novo Nordisk (Denmark) and Delta Pharma (Egypt), respectively. All chemicals and drugs were dissolved in saline for administration as mentioned in the experimental design.

### Animals

Twenty-eight male Sprague‒Dawley rats (7–8 weeks old) weighing 180–220 g were obtained from the animal facility of the Faculty of Pharmacy, Mansoura University, Egypt. They were allowed one week to adjust to the new experimental setting in typical cages. These settings included 12-h light and dark cycles, 30% to 60% humidity levels, a temperature of 22 ± 3 °C, free access to water, and a normal chow diet (~60% of calories from carbohydrates).

### Ethical approval and consent to participate

The animal care and experiments in this study were reviewed and received ethical approval by the “Research Ethics Committee” of the Faculty of Pharmacy at Mansoura University, Egypt (Code Number 2022-175) and Mansoura University Animal Care and Use Committee (MU-ACUC), Egypt, Code Number MU-ACUC (PHARM.R.25.09.61). We have complied with all relevant ethical regulations for animal use. These guidelines are based on the “Principles of Laboratory Animal Care” outlined in NIH publication No. 85-23, amended in 1985. Every possible effort was undertaken to reduce the distress experienced by the animals and to restrict the number of animals utilized.

### Experimental design

The rats were assigned to one of four groups (each group consisting of 7 male rats) via a random selection process:

- In the normal group, the rats were administered 0.2 mL of saline via intraperitoneal (i.p.) injection daily for 45 days.
- In the AlCl_3_ group, the rats were administered 70 mg/kg AlCl_3_ dissolved in 0.2 mL of saline (i.p.) once daily for 45 days^29,30^.
- In the AlCl_3_ + Lira group, the rats were administered 0.3 mg/kg Liraglutide dissolved in 0.2 mL saline subcutaneously twice daily (at 8:30 a.m., 30 min before AlCl3, and at 2 p.m.) for 45 days^31–33^.
- In the AlCl_3_ + done group: rats were administered 1 mg/kg Donepezil dissolved in 0.2 mL of saline (i.p.) once daily (at 8:30 a.m., 30 min before AlCl3) for 45 days^34^.

### Behavioral analysis

**Fig. 1:**
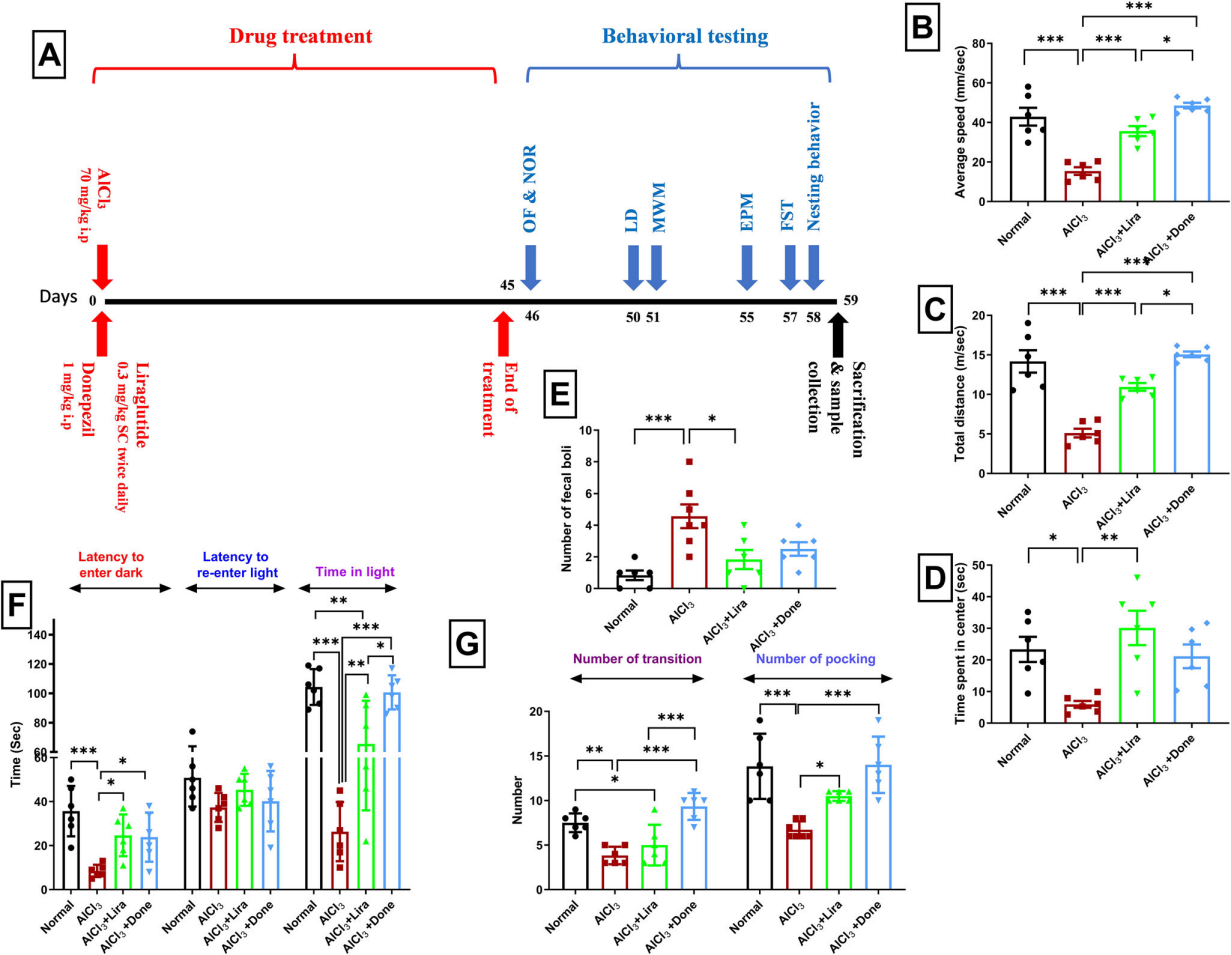
Liraglutide significantly ameliorated AlCl_3_-induced anxiety-like behavior. The timeline of the experimental design and behavioral analyses (**A**), the ameliorative effects of Liraglutide (Lira) and Donepezil (Done) on aluminum chloride (AlCl_3_)-induced Alzheimer’s disease in rats according to (**B**–**E**) open field and (**F**, **G**) light/dark tests. (*n* = 6 male rats). The data are presented as the means ± standard errors of the means (SEMs). Statistical analysis was performed via one-way ANOVA, followed by the Tukey multiple comparison test. ∗*P* < 0.05, ∗∗*P* < 0.01, and ∗∗∗*P* < 0.001. OF open field test, NOR novel object recognition test, LD light/dark test, MWM Morris water maze test, EPM elevated plus maze test, FST forced swimming test.

The behavioral tests were conducted by a single researcher exclusively during the light phase, specifically between 9:00 and 16:00 h. Trials were recorded via a high-resolution camera, and the resulting videos were examined by a skilled researcher unaware of the groups and treatments being studied. Fig. 1A illustrates the sequence of performance for various behavioral tests.

#### Open field (OF) test

According to Blanchard et al.^35^, anxiety-like behavior in rodents can be defined as a defensive response triggered by the presence of possible dangers. Defensive behaviors include evaluating potential risks and actively avoiding locations where the hazard may be present.

The test apparatus consisted of a vacant box with tall outside walls to prevent rat escape (60 × 60 × 40 cm). Test sessions of 5 min were videotaped, during which each animal was allowed to navigate the box unrestrictedly. A square region with dimensions of 30 × 30 cm was defined at the center of the image in the ToxTrac video-tracking system, which is an automated open-source software for tracking animals^36^.

The analysis compared the time spent in the core area versus the periphery, indicating the risk-assessing behavior of the rats versus anxiety. Additionally, the total distance traveled in meters and the average speed in millimeters per second were examined as measures of locomotor activity. After every testing session, the box floor and walls were consistently cleaned with a 10% alcohol solution to remove any potential smell clues.

#### Light/dark (LD) test

The test depends on rodents’ inherent aversion to novel, well-lit environments ^26^. The apparatus consisted of a larger open, well-lit compartment (30 × 30 × 38 cm) and a smaller enclosed, dark compartment (30 × 19 × 38 cm). Both compartments were connected by a small opening placed downward.

Test sessions of 5 min started with the animals positioned in the central area of the well-lit compartment, with their heads pointing away from the opening. As primary measures of anxiety, latency to first exit light, latency to return to light, time spent in light, and number of transitions between the two compartments were analyzed. The number of head-outs into the well-lit compartment was considered a measure of the animals’ risk-assessing behavior.

#### Novel object recognition (NOR) test

As previously explained by Cutuli et al.^37^, the NOR test consists of 3 min each: the habituation, training and test phases, which last 5 min. Using the box previously described for the OF test, we considered the 5 min of the OF test as the habituation phase, after which the rats were transferred to their home cages, allowed 3 min, and then started the training phase directly. During the training phase, the rats were given 5 min to investigate two identical objects. One hour later, a test phase was conducted using one familiar object and a novel object, and the rats were allowed to explore both objects for another 5 min. The duration of the rats’ exploration of the two objects during the test phase was recorded via a video camera and examined by an experienced observer. The discrimination index was computed via the following formula:

$$	{\mathrm{Discrimination}}{\mathrm{index}}=\\ 	\frac{\,{\mathrm{TimespentinvestigatingNovelobject}}{-}{\mathrm{TimespentinvestigatingOldobject}}}{{\mathrm{Totaltimespentexploringbothobjects}}}$$

#### Morris water maze (MWM) test

The test apparatus consisted of a circular pool with a black color, a height of 45 cm and a diameter of 150 cm. The pool was filled with white opaque water (24 ± 2 °C, 30 cm in depth) and partitioned into four quadrants by intersecting filaments at a 90° angle. These quadrants are labeled as follows: northeast (NE), southeast (SE), southwest (SW), and northwest (NW). An invisible circular escape platform with a diameter of 10 cm was positioned in the southeastern quadrant, 2 cm below the water surface. It remained in that quadrant throughout the entire duration of the experiment^38^. Three signs were strategically positioned on the walls surrounding the pool to instruct the rats to utilize them to find a more direct route toward the submerged escape platform.

First, four consecutive memory acquisition trials were performed, each lasting 120 s. There was a 5-min break between each trial. The trials used a set of semirandom start points (N, W, NE, and SW). The trial was terminated when the rat sprang onto the concealed platform. Anytime a rat failed to find the platform, it was gently guided to the platform and left on for 20 s. A memory retention trial was undertaken 24 h following the acquisition trials, and a trained observer recorded the escape latency (the time it took to jump onto the hidden platform) during this trial^39^.

#### Elevated plus-maze (EPM) test

The test apparatus consisted of four arms arranged as a “+” maze positioned 50 cm above the ground. The arms were connected at the center by an open square platform (10 × 10 cm). The two arms were open, meaning they had no walls (50 × 10 cm). The other two arms were closed, meaning they had walls (50 × 10 × 40 cm high).

On day 55, a trial for memory acquisition was conducted following the same procedure as described by Morales-Delgado et al.^40^. Each animal was placed at the end of an open arm facing away from the center. The time taken until the animal entered one of the closed arms (acquisition-transfer latency) was recorded by an experimenter blinded to the study design. The rats were given 20 s to navigate the maze before returning to their home cages. If a rat did not enter the closed arm within the 90-s time limit, it was carefully directed into the closed arm, and the acquisition-transfer latency for that rat was recorded as 90 s.

Twenty-four hours later, a second trial to evaluate memory retention was conducted and the retention-transfer latency was recorded. A longer retention-transfer latency indicated poor memory retention. The maze was sanitized with 10% ethanol between each animal.

#### Forced swimming test (FST)

The test apparatus comprised a cylindrical container made of glass (20 cm diameter × 60 cm height) filled with water (24 ± 2°C, 25 cm high). Using a video camera installed at the front of the apparatus, rat behavior was recorded for 5 min in terms of struggling/immobility time. Struggling was defined as any intentional attempt to escape. Moreover, the immobility time was recorded by an experienced observer when the animal floated with minimal movement to keep its head above the water level. The total immobility time was recorded as a marker of depressive behavior^41^.

#### Nesting behavior

The rats were housed individually with approximately 100 g of regular bedding and 2.5 g of nesting material, consisting of 5–7 cm strips of clean, shredded white paper, for 24 hours. The nesting score, ranging from 1 to 5 and derived from Deacon et al.^42^, was employed to evaluate nesting behavior. 1 = strips were present in various locations within the cage; 2 = more than half of the strips were scattered and not utilized for nesting; 3 = a noticeable nest was built, but up to half of the strips remained scattered; 4 = more than 90% of the strips were used for the nest, with only a few pieces of material remaining scattered; 5 = all strips were utilized to create a recognizable nest.

### Dissection and tissue preparation

Twenty-four hours after the final behavioral assessment, the rats were euthanized with a combination of 50 mg/kg ketamine and 10 mg/kg xylazine IP. The brains were rapidly removed and washed with ice-cold phosphate-buffered saline (pH 7.4) (PBS) (Biodiagnostic Co., Giza, Egypt). Under the dissecting microscope, the left and right hemispheres were separated at the midsagittal plane. The hippocampi and frontal cortices were then carefully dissected as described previously^43^. Both samples were divided into two (left and right) portions: the left portion was preserved in 10% neutral buffered formalin for histopathological and immunohistochemical investigation, while the right portion was homogenized in PBS and centrifuged. The resulting supernatant was stored at −80 °C for subsequent biochemical analysis.

### Biochemical analysis

The supernatants of cortical homogenates were utilized to assess reduced glutathione (GSH, # GR2511) and malondialdehyde (MDA, #MD2529) levels via commercially available colorimetric kits (Biodiagnostic Co., Giza, Egypt). The absorbances were measured via an 850 UV/Vis double-beam spectrophotometer (Jenway, UK).

### Neurochemical analysis

The hippocampal homogenate’s supernatant was utilized to assess the acetylcholinesterase (AChE) (#BYEK3333) activity via a commercially available colorimetric kit (Chongqing Biospes Co., Ltd., Chongqing, China). The absorbances were measured via an 850 UV/Vis double-beam spectrophotometer (Jenway, UK).

### ELISA

The supernatant of the hippocampal tissue homogenate was used to assess the OxLDL (# BYEK3557) (Chongqing Biospes Co., Ltd., Chongqing, China), LPA (#BZEK1313) (Chongqing Biospes Co., Ltd., Chongqing, China), GLP-1 receptor (#ER0418) (FineTest, China), BACE1 (#RTEB0425) (AssayGenie, China) and LPAR1 (#RTEB1695) (AssayGenie, China) levels via ELISA kits with the help of a BioTek ELX800 (USA) microplate reader for measuring absorbances.

### Histopathological analysis

#### Hematoxylin and Eosin (HE) staining

The second portions of each cerebral cortex and hippocampus were immersed in formalin buffer for 24 h to preserve their structure. They were processed, enclosed in paraffin blocks, and cut into 5 μm thick sections. The obtained tissue sections were subjected to H&E staining and photographed via a digital camera attached to a BX41 microscope (Olympus Corporation, Tokyo, Japan).

### Modified Bielschowsky silver staining

Modified Bielschowsky silver staining was utilized to evaluate the hallmark lesions of AD, Aβ plaques and neurofibrillary tangles^44^. Briefly, 40 μm coronal brain sections were rehydrated and coated with 20% AgNO3 for 20 min without light. The sections were subsequently washed with distilled water and immersed in the ammonium silver solution for 10 min. Finally, the slides were submerged in water containing ammonia and then processed.

### Immunohistochemical analysis

For immunohistochemistry, 5-µm-thick sections were deparaffinized and rehydrated in a graded series of alcohol. A 3% hydrogen peroxide in 10% methanol solution was used to eliminate internal peroxidase interference. The sections were then placed in blocking serum, which consisted of a 5% standard goat serum solution in 0.3% Triton with PBS. Afterwards, the slides were treated with rabbit BACE1 polyclonal primary antibody (#CSB-PA008418, Cusabio, China; 1:200 dilution), LPAR1 polyclonal primary antibodies (#NBP1-03363SS, NovusBio, USA; 1:200 dilution), mouse monoclonal anti-Bcl-2 (#61-0005, Genemed Biotechnologies, Inc., USA; 1:200 dilution), and rabbit BAX, Tau and amyloid precursor protein polyclonal antibodies (#A12009, #A1103, A17911, ABclonal, USA, respectively; 1:200 dilution) for 2 h at room temperature according to the manufacturer’s protocol. The unbound primary antibody was washed away with PBS; then, the slides were treated with the secondary antibody (biotinylated goat anti-rabbit antibody, 1:500) for an hour at room temperature. Finally, color was developed via diaminobenzidine (1:1000) incubation for 10 min at room temperature.

### Immunopathological analysis

Protein expression in the cerebral cortex and hippocampal regions was quantified as the percentage of immunopositive cells expressing BACE1, LPAR1, Bcl-2, BAX, Tau, or amyloid precursor protein. At least 6 random microscopic fields per group were analyzed via the image analysis software QuPath^45^. The burden of NFTs (quantified as the percentage of brown intracellular argyrophilic staining relative to the total field area) and amyloid plaques (quantified as the number of brown extracellular rounded accumulations per visual field) was analyzed using FIJI/IMAGE J v1.54i software.

### Statistics and reproducibility

The statistical analysis was conducted using SPSS v 27 (IBM Inc., Armonk, NY, USA) and GraphPad Prism 9. Charts were constructed via the GraphPad Prism 9 program, and the data were expressed as the means ± standard errors of the means (SEMs). Following the use of the Shapiro‒Wilk normality test to assess normality, data were subjected to one-way analysis of variance (ANOVA) and further via Tukey’s post-hoc testing for comparisons between four groups. In each separate group, the exploration time spent with novel versus old objects in the NOR test was compared using a paired samples t-test. The nesting behavior scores were analyzed via nonparametric analyses (Kruskal‒Wallis ANOVA followed by Dunn’s–Bonferroni test). All tests were two-tailed, with statistical significance established at *α* = 0.05. Each group consisted of six to seven biological replicates (*n* = 6–7 male rats). All experiments were replicated once, and each measurement corresponds to an independent animal.

Sample size was determined based on prior studies using the MWM in similar models^46,47^. Consistent with the previously reported robust effect of Liraglutide in AlCl₃-induced memory impairments^48^, post-hoc power analysis for the primary behavioral endpoint (escape latency in the MWM) yielded an effect size of Cohen’s *f* = 3.364; indicating that the study was fully powered to detect differences among the means of the different 4 groups (*η*^2^ = 0.919 at *α* = 0.05 for *n* = 6 per group). Power analysis was performed using the F-test for ANOVA in G*Power software (version 3.1.9.7).

### Reporting summary

Further information on research design is available in the Nature Portfolio Reporting Summary linked to this article.

## Results

### Liraglutide significantly improved the anxiety-like behavior induced by AlCl_3_

In the OF test, male rats display a natural preference for the periphery of the OF box, thigmotaxis, and a tendency to remain close to the box walls because of their innate fear of potential threats.

As an indicator of increased anxiety and impaired risk-taking behaviors, the time spent in the central area of the box was significantly lower in the AlCl_3_ group (*P* < 0.05) than in the normal group (Fig. 1D). Impaired locomotor activity was also evident in the AlCl_3_ group, as they displayed a marked decrease in their average speed and total distance moved (*P* < 0.001 vs. normal) (Fig. 1B, C). Unlike Donepezil, Liraglutide treatment was associated with lower anxiety levels and a greater tendency for risk-taking, as evidenced by the marked increase in the time spent in the center compared with those in the AlCl_3_ group (*P* < 0.005) (Fig. 1D). Locomotor activity (average speed and total distance traveled) was also significantly increased in the Liraglutide group (*P* < 0.001 vs. the AlCl_3_ group) (Fig. 1B, C).

In the LD test, anxiety-like behavior is characterized by early leaves to the dark compartment, decreased time spent in the well-lit compartment, and increased transitions between the light and dark compartments. This profile was observed within the AlCl_3_ group. Our data revealed that the AlCl_3_ group tended to leave the dark (*P* < 0.001) rapidly, spend markedly less time in the light (*P* < 0.005), and transit less between the light and dark (*P* < 0.001) than the normal group did (Fig. 1F, G). However, Liraglutide treatment significantly increased the latency to leave to the dark (*P* < 0.05) and the time spent in light (*P* < 0.005). In addition to enhanced risk-taking behavior, the number of head-outs (poking) also markedly increased (*P* < 0.05) in the AlCl_3_ + Lira group (Fig. 1F, G).

### Liraglutide significantly reversed the impairment of recognition memory caused by AlCl_3_

**Fig. 2:**
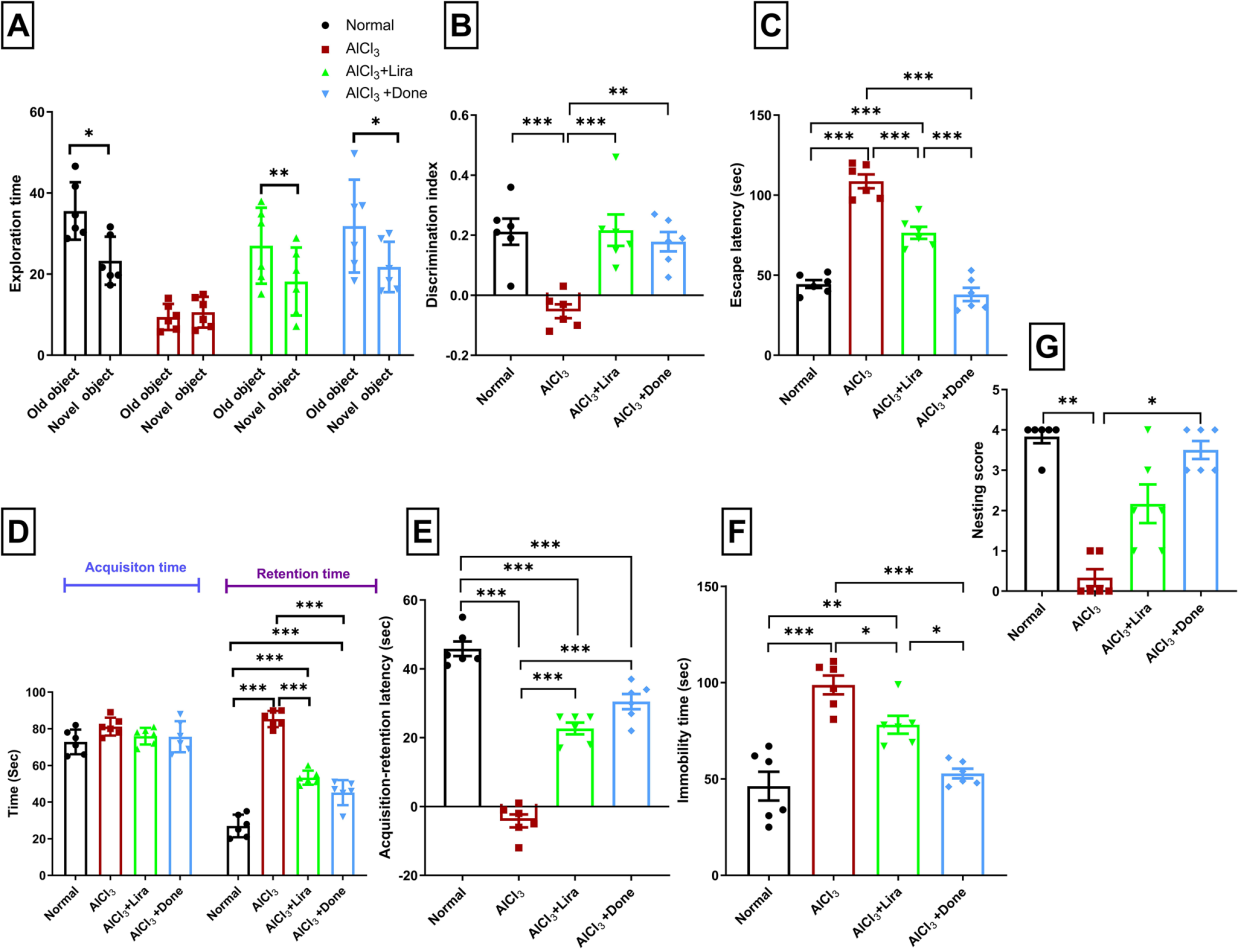
Liraglutide significantly improved AlCl_3_-induced deficits in recognition memory, long-term spatial learning, memory, depression-like behavior and restored nesting behavior. The ameliorative effects of Liraglutide (Lira) and Donepezil (Done) on aluminum chloride (AlCl_3_)-induced Alzheimer’s disease in rats via **A**, **B** novel object recognition, **C** Morris water maze **D**, **E** elevated plus maze tests, **F** forced swimming and **G** nesting behavior tests. (*n* = 6 male rats). The data are presented as the means ± standard errors of the means (SEMs). Except for the analysis of (**A**) the exploration time in each separate group, which was performed via a paired samples t test, statistical analysis was performed via one-way ANOVA, followed by the Tukey multiple comparison test. ∗*P* < 0.05, ∗∗*P* < 0.01, and ∗∗∗*P* < 0.001.

In the NOR test, the normal, Liraglutide, and Donepezil groups exhibited a significant inclination toward the novel object vs. the familiar object, unlike the AlCl_3_ group (Fig. 2A).

When the discrimination index was calculated, a marked decrease in recognition memory was noted in the AlCl_3_ group compared with the normal group. The AlCl_3_ group spent nearly the same amount of time sniffing the novel and familiar objects (*P* > 0.005) (Fig. 2A). Interestingly, treatment with Liraglutide resulted in a significant increase in recognition memory and efficiently reduced memory impairment (*P* < 0.001) (Fig. 2A, B).

### Liraglutide significantly improved AlCl_3_-induced impairments in long-term spatial learning and memory

Chronic AlCl_3_ administration resulted in significantly prolonged escape latency compared to the normal group. On the other hand, Liraglutide treatment significantly decreased escape latency when compared to the AlCl_3_ group (*P* < 0.001) (Fig. 2C).

In the EPM test, AlCl_3_ administration had similar adverse effects on long-term memory, as it caused marked delay in the retention-transfer latency compared with the normal group (*P* < 0.001) (Fig. 2E). Compared with Donepezil treatment, Liraglutide significantly (*P* < 0.001) improved the retention of memory in the AlCl_3_-induced Alzheimer’s rat model (Fig. 2D, E).

### Liraglutide significantly prevented AlCl_3_-induced depression-like behavior

Compared with the normal group, the AlCl_3_ group exhibited a longer duration of immobility (*P* < 0.001) as well as a significant decrease in the nesting score (*P* < 0.005). Compared with the AlCl_3_ group, Liraglutide significantly decreased the duration of immobility (*P* < 0.05) (Fig. 2F), although it did not markedly improve the nesting behavior (*P* > 0.05) (Fig. 2G).

### Liraglutide significantly prevented AlCl_3_-induced cerebral and hippocampal histopathological alterations

**Fig. 3:**
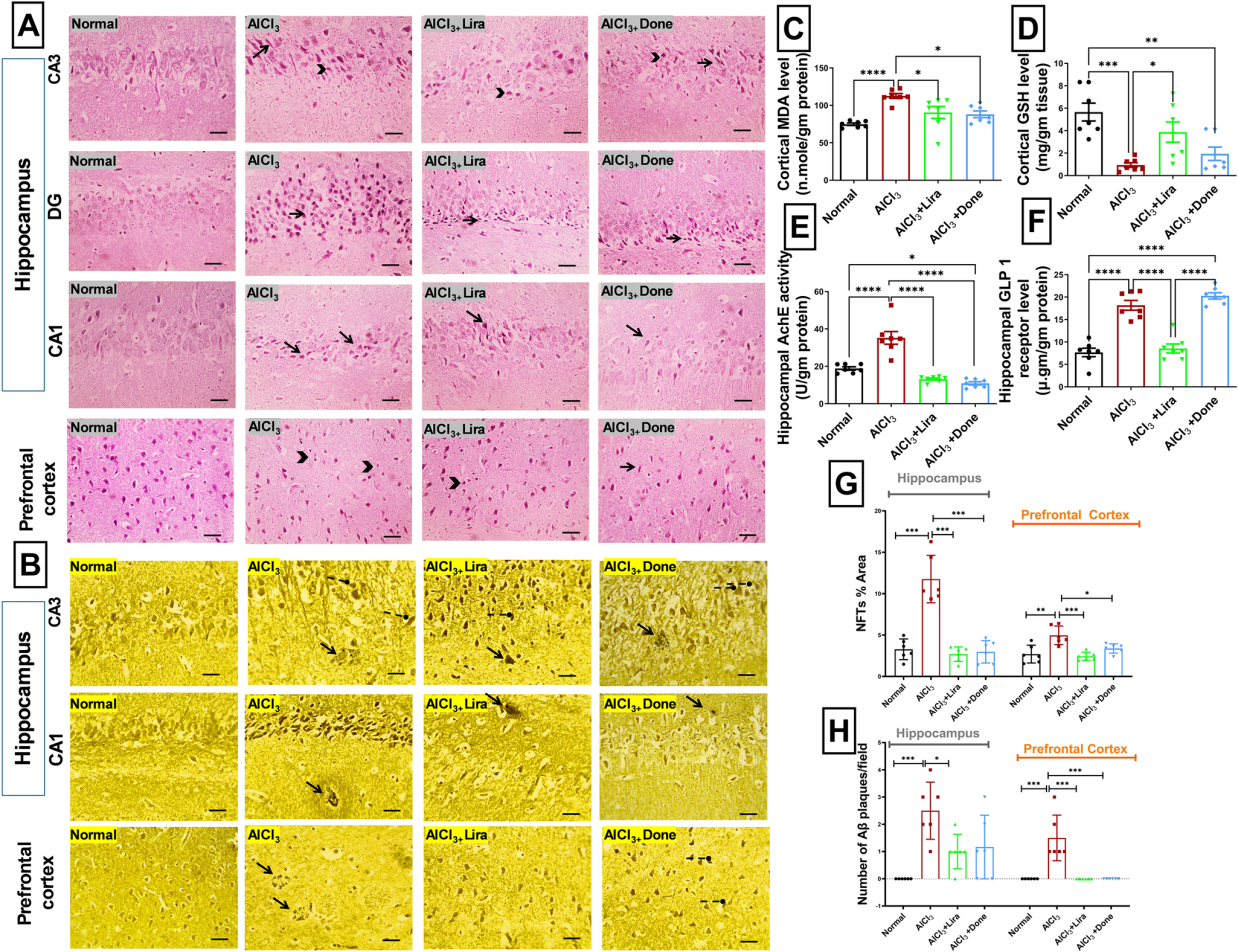
Liraglutide significantly prevented AlCl_3_-induced neuronal degeneration, oxidative stress and neurochemical dysregulation in an Alzheimer’s disease rat model. Representative images of H&E-stained sections. Hippocampal CA3 region, dentate gyrus (DG), Hippocampal CA1, prefrontal cortex (**A**). Microscopic sections from the Alzheimer’s disease group revealed marked neuronal loss, degeneration (deeply stained shrunken cytoplasm; arrows), and apoptosis (small dark pyknotic nuclei; arrowheads). Compared with those in the Donepezil-treated group, the number of neurons in the Liraglutide-treated group increased, and mild to moderate neuronal degeneration was detected (400×, scale bar 50 µm). Representative images of Bielschowsky silver-stained sections. Hippocampal CA3 region, Hippocampal CA1 region, Prefrontal cortex (**B**). Sections from the Alzheimer’s group show multiple large, rounded amyloid plaques (arrows) outside the cells. Filamentous neurofibrillary tangle-like structures (round-ended arrows) are also observed inside the cells. Compared with Donepezil treatment, Liraglutide treatment markedly decreased the number of neurofibrillary tangle-like structures in the hippocampal sections (400×, scale bar 50 µm). The ameliorative effects of Liraglutide (Lira) and Donepezil (Done) against aluminum chloride (AlCl_3_)-induced Alzheimer’s disease in rats on cortical malondialdehyde (MDA) levels (**C**, *n* = 7 male rats) reduced glutathione (GSH) levels (**D**, *n* = 7 male rats), hippocampal acetylcholinesterase (AChE) activity (**E**, *n* = 7 male rats) and hippocampal glucagon-like peptide-1 (GLP-1) receptor level (**F**, *n* = 7 male rats), the NFTs % Area in the silver stained sections for hippocampal and cortical areas (**G**, *n* = 6 male rats) and the number of Aβ plaques/field in the silver-stained sections for hippocampal and cortical areas (**H**, *n* = 6 male rats). The data are presented as the means ± standard errors of the means (SEMs). Statistical analysis was performed via one-way ANOVA, followed by the Tukey multiple comparison test. ∗*P* < 0.05, ∗∗*P* < 0.01, and ∗∗∗*P* < 0.001.

AD-related neuropsychiatric symptoms, including cognitive deficits and emotional dysregulation, are most likely the result of damage to the hippocampus and prefrontal cortex. H&E-stained sections of cortices and hippocampi from the AlCl_3_ group exhibited marked neuronal loss, degeneration, and apoptosis. Irregularly damaged cells with indistinct cell boundaries and deeply stained shrunken cytoplasm were noted. On the other hand, increased neuronal numbers and mild to moderate neuronal degeneration were observed in the cortical sections of the Donepezil- and Liraglutide-treated groups (Fig. 3A).

Similarly, the hippocampal sections from the Donepezil- and Liraglutide-treated groups presented circular, closely arranged neurons with intact nuclear membranes. Only mild to moderate neuronal degeneration was observed in the Liraglutide- and Donepezil-treated groups (Fig. 3A). Apparent differences in neuronal morphology and size across groups reflect pathological alterations induced by AlCl₃ and the neuroprotective effects of liraglutide and donepezil. Hippocampal neurones exhibit significant vulnerability to oxidative stress and amyloid accumulation, resulting in shrinkage, vacuolation, and the degeneration of pyramidal cells in the AlCl₃ group, whereas the therapy groups demonstrate a partial recovery of cytoarchitecture.

### Liraglutide significantly ameliorated AlCl_3_-induced oxidative stress and neurochemical dysregulation

Moreover, modified Bielschowsky silver staining revealed multiple rounded amyloid plaque-like structures in the cortical sections of the AlCl_3_ group (Fig. 3B), which were improved by Liraglutide and Donepezil treatment. Filamentous neurofibrillary tangle-like structures were also observed inside the pyramidal neurons of the CA3 and CA1 hippocampal region in the AlCl_3_ group (Fig. 3B). Notably, the expression of these structures was ameliorated by Liraglutide treatment. AlCl_3_ caused a significant increase in NFTs % Area in both hippocampal and cortical areas (Fig. 3G), along with a significant increase in the number of Aβ plaques/field in both hippocampal and cortical areas (Fig. 3H). Liraglutide significantly decreased both NFTs % Area and number of Aβ plaques/field in both hippocampal and cortical areas compared to AlCl_3_ group (Fig. 3G, F, respectively). As well as Donepezil significantly decreased both NFTs % Area in both hippocampal and cortical areas and number of Aβ plaques/field in both cortical area only, compared to AlCl_3_ group (Fig. 3G, F, respectively).

Compared with the normal group, the AlCl_3_ group presented a significantly decreased cortical GSH level (Fig. 3D) with a significant increase in the cortical MDA level (Fig. 3C), which indicates a decreased antioxidant level with increased lipid peroxidation in the cortical tissue. Compared with AlCl_3_, Liraglutide successfully increased the cortical GSH level (Fig. 3D). Compared with AlCl_3_, Liraglutide and Donepezil significantly decreased the cortical MDA level (Fig. 3C).

Moreover, AlCl_3_ caused neurotransmitter dysregulation, as it caused a significant increase in hippocampal AChE activity compared with that in the normal group. On the other hand, Liraglutide and Donepezil significantly decreased hippocampal AChE activity (Fig. 3E) compared with those in the AlCl_3_ group.

In addition, as Liraglutide is a GLP-1 receptor agonist, we investigated the effects of AlCl_3_ alone and in combination with Liraglutide and Donepezil on the hippocampal GLP-1 receptor. Compared with the normal group, the AlCl_3_ and AlCl_3_ + Done groups presented a significant increase in the hippocampal GLP-1 receptor. In contrast, the AlCl_3_ + Lira group demonstrated a significant decrease in the hippocampal GLP-1 receptor compared with the AlCl_3_ group (Fig. 3F).

### Liraglutide significantly prevented AlCl_3_-induced upregulation of the oxLDL/LPA/LPAR pathway

**Fig. 4:**
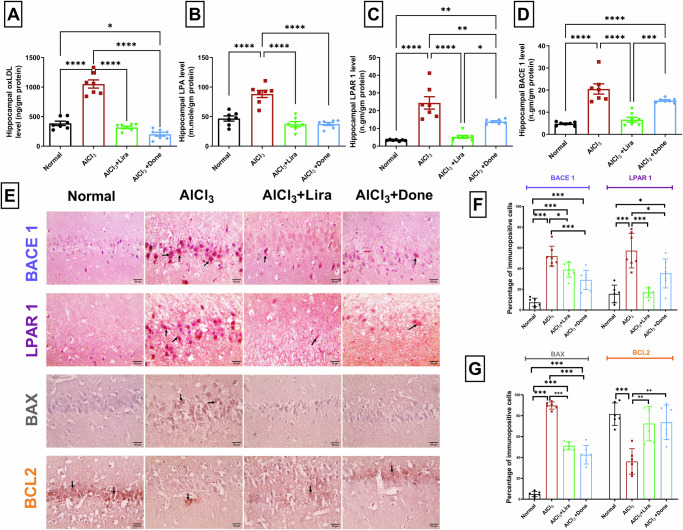
Liraglutide significantly prevented AlCl_3_-induced upregulation of the oxLDL/LPA/LPAR1 pathway. The ameliorative effects of Liraglutide (Lira) and Donepezil (Done) against aluminum chloride (AlCl_3_)-induced Alzheimer’s disease in rats on hippocampal oxidized-low-density lipoprotein (oxLDL) levels (**A**, *n* = 7 male rats), lysophosphatidic acid (LPA) levels (**B**, *n* = 7 male rats), Lysophosphatidic acid receptor (LPAR1) level (**C**, *n* = 7 male rats), β-secretase (BACE1) level (**D**, *n* = 7 male rats), Immunohistochemical images of the hippocampal CA3 region immunolabeled with BACE1 and LPAR1, BAX and Bcl2 antibodies (**E**). (400×, scale bar 50 µm), percentages of cells immunopositive for BACE1and LPAR1 (**F**, *n* = 7 male rats), **G** percentages of cells immunopositive for BAX and BCL2 (**G**, *n* = 6 male rats). The data are presented as the means ± standard errors of the means (SEMs). Statistical analysis was performed via one-way ANOVA, followed by the Tukey multiple comparison test. ∗*P* < 0.05, ∗∗*P* < 0.01, and ∗∗∗*P* < 0.001.

Compared with the normal conditions, AlCl_3_ significantly increased the hippocampal levels of oxLDL, LPA, LPAR1, and BACE1. Compared with AlCl_3_, Liraglutide and Donepezil significantly decreased the hippocampal levels of oxLDL, LPA, and LPAR1. In addition, Liraglutide significantly decreased the hippocampal levels of BACE1 compared with those in the AlCl_3_ group (Fig. 4A–D). Moreover, tissue expression of both BACE1 and LPAR1 confirmed these results, as the hippocampal sections from the AlCl_3_ group were markedly increased compared with those from the normal group. In contrast, Liraglutide and Donepezil treatment significantly decreased hippocampal BACE1 and LPAR1 expression compared with that in the AlCl_3_ group (Fig. 4E, F).

### Liraglutide significantly prevented AlCl3-induced upregulation of BAX, amyloid precursor protein and Tau in addition to downregulation of BCL-2

To elucidate the underlying mechanism, the role of apoptosis in AlCl_3_-induced AD was investigated. Compared with the normal group, AlCl_3_ caused a significant increase in hippocampal BAX tissue expression and a significant decrease in BCL-2 tissue expression. Liraglutide and Donepezil treatment significantly ablated these effects, as they significantly decreased the hippocampal tissue expression of BAX and significantly increased the tissue expression of BCL-2 compared with those in the AlCl_3_ group (Fig. 4E, G).

**Fig. 5:**
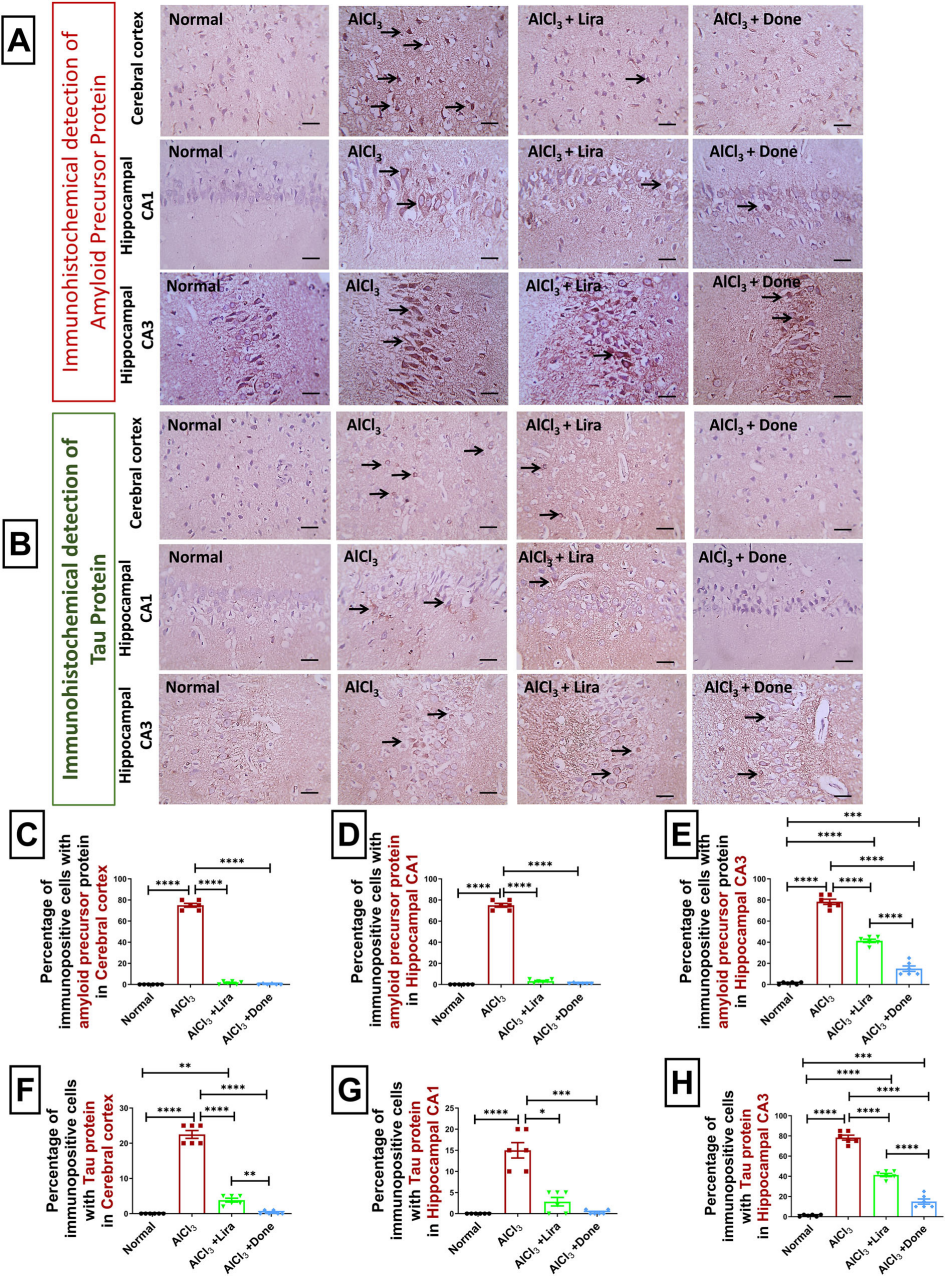
Liraglutide significantly prevented AlCl_3_-induced upregulation of the amyloid precursor protein and Tau proteins. Immunohistochemical images of the cerebral cortex, hippocampal CA1 and hippocampal CA3 regions immunolabeled with amyloid precursor protein (**A**) (400×, scale bar 50 µm) and Tau (**B**) antibodies. (400×, scale bar 50 µm), percentages of cells immunopositive for amyloid precursor protein in cerebral cortex (**C**), hippocampal CA1 (**D**), and hippocampal CA3 (**E**). Percentages of cells immunopositive for Tau in cerebral cortex (**F**), hippocampal CA1 (**G**) and hippocampal CA3 (**H**). (*n* = 6 male rats). The data are presented as the means ± standard errors of the means (SEMs). Statistical analysis was performed via one-way ANOVA, followed by the Tukey multiple comparison test. ^∗^*P* < 0.05, ^∗∗^*P* < 0.01, and ^∗∗∗^*P* < 0.001.

To state more, the Alzheimer progression and the neurodegeneration effect of AlCl_3_, as well as the neuroprotective effects of Liraglutide and Donepezil, the immunohistochemical expression of amyloid precursor protein and Tau has been evaluated. AlCl_3_ markedly increased positive brown neuronal expression of amyloid precursor protein compared to the normal group in the cortex (Fig. 5A, C) and hippocampal CA1 (Fig. 5A, D) and CA3 (Fig. 5A, E). On the other hand, Liraglutide and Donepezil caused a marked decrease in positive brown neuronal expression of amyloid precursor protein compared to the AlCl_3_ group in the cortex (Fig. 5A, C) and hippocampal CA1 (Fig. 5A, D) and CA3 (Fig. 5A, E).

Moreover, the immunohistochemical expression of Tau came in accordance with these results as AlCl_3_ markedly increased positive brown neuronal expression of Tau compared to the normal group in the cortex (Fig. 5B, F) and hippocampal CA1 (Fig. 5B, G) and CA3 (Fig. 5B, H). On the other hand, Liraglutide and Donepezil caused marked decrease in positive brown neuronal expression of Tau protein in cortex (Fig. 5B, F) and hippocampal CA1 (Fig. 5B, G) and CA3 (Fig. 5B, H) compared to the AlCl_3_ group.

## Discussion

AD represents a sporadic, devastating neurodegenerative disorder. Increasing age is the most well-stated risk factor. However, the precise underlying mechanism is poorly understood. Owing to the lack of a cure, understanding the underlying pathogenic mechanism of AD and finding efficacious therapeutic approaches are critical.

In the current study, we used Donepezil as a reference drug, as it is an FDA-approved drug for mild to severe AD. This is related to its modulation of different neurotransmitter systems, such as the reduction of the pro-inflammatory cytokine, decreasing the level of amyloid precursor protein, modulating the cholinergic effects and oxidative stress. Studies have stated the neuroprotective effects of Donepezil in vivo or in vitro experiments in AD models, suggesting its ability to counter the progressive degeneration of brain neurons and the hippocampal atrophy, maintaining the functional brain activity^20,49–51^.

Due to the pathophysiological commonalities between DM and neurodegenerative diseases, investigating the repurposing of anti-diabetic drugs for AD has gained a lot of interest. Several animal and clinical studies focused on one of the antidiabetic drugs, metformin. The literature is conflicting, with a meta-analysis indicating that metformin use failed to reduce the risk of AD development and increased PD risk^52^. As well, observational human studies are conflicting, but those with better study designs suggest that metformin use in persons with type 2 diabetes is associated with a lower risk of dementia^53^. Moreover, Pioglitazone, a peroxisome proliferator-activated nuclear receptor γ (PPARγ) agonis antidiabetic drug showed neuroprotective effects in animal models of AD^54,55^. While, unfortunately, in 2019, a large clinical trial was therefore needed to assess its effectiveness in non-diabetes mellitus AD patients or those at risk and was deemed unsuccessful^56^.

In the present study, chronic exposure to AlCl_3_ aggravated anxiety-like behaviors, impaired both recognition and long-term spatial memories, and aggravated depression-like behavior. Liraglutide significantly ameliorated these behavioral abnormalities, indicating the promising potential of Liraglutide in ameliorating AD. The benefits of Liraglutide for long-term spatial learning and memory in the MWM test are consistent with recent publications^16,57^. Moreover, the findings of Duarate et al*.* are in line with our results regarding anxiety-like behavior and long-term spatial learning and memory in the OF and MWM tests, respectively, in a different AD model in female mice^18^.

Histopathological examination of the cerebral cortex and hippocampus (pyramidal layer and dentate gyrus) confirmed the results of the behavioral tests. The AlCl_3_ group presented a decrease in the number of neurons due to loss, degeneration, and apoptosis, with multiple amyloid plaques in different regions. However, the groups treated with Liraglutide and Donepezil showed significant improvement. These results agree with Carranza-Naval et al*.*^57^ who studied their effect in a mixed murine model of AD and type 2 diabetes.

The cerebral GSH and MDA levels are consistent with the relationship between AD and oxidative stress; AlCl_3_ depletes GSH and increases lipid peroxidation (MDA level). Liraglutide successfully maintained normal levels of GSH and MDA, while Donepezil maintained only the normal level of MDA AD’s effect on GSH aligns with the results reported by Christopher et al.^58^ in the AD model in transgenic mice. The effect of Liraglutide on MDA level was in accordance with an in vitro study on human neuroblastoma SH-SY5Y cells^59^. Moreover, the results of Donepezil on MDA level were in accordance with a previous study investigating the effect of Donepezil on scopolamine-induced amnesia in rats^60^. These findings have emphasized the involvement of oxidative stress and increased reactive oxygen species (ROS) in plaque deposition and neuronal and synaptic dysfunction, subsequently leading to AD development^61,62^.

In addition to the role of oxidative stress in mediating AD pathogenesis, the cholinergic system plays a crucial role. Chronic Al exposure in rodent models has been consistently linked to cholinergic dysfunction, which eventually led to neurocognitive impairments^63^. Donepezil, as an AchE inhibitor, holds the potential to enhance cholinergic transmission and improve major cognitive domains of memory and learning. Indeed, a large body of evidence supports the use and efficacy of Donepezil as a positive comparator in the AlCl3-induced AD model^64,65^. Owing to its cholinergic effects, Donepezil is one of the first-line pharmacotherapies for mild to moderate AD^66,67^. Our results revealed that Liraglutide significantly decreased AChE, similar to Donepezil, compared with that in the AlCl_3_ group. To our best knowledge, this study is the first study to elucidate the effect of Liraglutide on AChE activity. The investigation of the effect of AlCl_3_ alone and in combination with Liraglutide and Donepezil on hippocampal GLP-1 receptor showed that AlCl_3_ alone and in combination with Donepezil upregulated the GLP-1 receptor. Lee et al.^68^ stated the decrease of GLP-1 receptor expression in Experimental Autoimmune Encephalomyelitis, which came in disagreement with our results. The increase of GLP-1 receptor in the AlCl_3_ group may be a compensatory response to neurotoxicity. On the other hand, Liraglutide caused a significant decrease in GLP-1 receptor compared to the AlCl_3_ group, which may be due to the binding of Liraglutide to the GLP-1 receptor and binds its activation, triggering homologous desensitization and receptor internalization, causing its decreased level due to the chronic exposure to Liraglutide^69,70^. On the other hand, the neuroprotective effect of Donepezil is mainly mediated through its inhibition of acetylcholinesterase; thus, it does not interfere with GLP-1 receptor dynamics. In addition, we should acknowledge that ELISA-based quantification of GLP-1 receptor presents inherent challenges. While the kit used in this study was validated by the manufacturer with acceptable sensitivity, specificity, and reproducibility parameters, potential limitations include restricted dynamic range, possible cross-reactivity with related receptor isoforms, and variable sensitivity depending on sample type. Furthermore, ELISA measures total receptor protein and may not fully discriminate between active and inactive receptor forms^71^. These limitations should be considered when interpreting the results and warrant cautious extrapolation.

To elucidate the underlying molecular mechanisms that affect both oxidative stress and the cholinergic system, we investigated the oxLDL/LPA/LPAR1/BACE1 pathway. Chronic AlCl_3_ exposure significantly upregulated the oxLDL/LPA/LPAR1/BACE1 pathway. OxLDL, the source of LPA, mediates many of oxLDL’s downstream effects^72^. LPA signals through at least six G protein-coupled receptors, with LPAR1 being one of the best-characterized. LPA-LPAR1 signaling activates various intracellular pathways, including MAPK and Rho, influencing cell proliferation, migration, and survival^73^. In addition, LPA has been stated to increase the expression of BACE1, which increases Aβ production^74^. Moreover, Lipids extracted from oxLDL are stated to directly increase BACE1 activity and Aβ production in neuronal cells^72^.

Dysfunction of LPA metabolism has been stated to result in neuroinflammation^75,76^, neuronal apoptosis^77^, Aβ peptide aggregation^26,78^, and BACE1 upregulation^77^. This came in agreement with our results as AD caused an upregulation of LPA/ LPAR1/ BACE1 in addition to apoptosis (increase in BAX and decrease in BCL2 tissue expressions). In addition, LPA is the major bioactive component of oxLDL, which can disrupt blood-brain barrier function and cause AD-related cellular events. This agrees with our results of the increase in oxLDL level in experimentally induced AD. Thus, AlCl_3_ caused upregulation of LPA, which caused upregulation of oxLDL, LPAR1, and BACE1, causing oxidative stress, apoptosis, and Aβ peptide aggregation. These result in AchE upregulation, histopathological alteration of cortical and hippocampal structure, and AD pathogenesis. Thus, modulating the oxLDL/LPA/LPAR1/BACE1 pathway represents a standard target for ameliorating AD.

Both Liraglutide and Donepezil successfully modulated the oxLDL/LPA/LPAR1/BACE1 pathway and successfully prevented AD in rats. The repurpose of Liraglutide in AD treatment may provide a new effective treatment for AD with lower cost and time needed for new discovery. Liraglutide successfully ameliorated LPAR1, oxLDL, and BACE1, showing antioxidant and anti-apoptotic abilities. The effect of Liraglutide on oxLDL came in accordance with Wang and Yang who stated that Liraglutide reduces oxLDL-induced oxidative stress and lipid accumulation in macrophage-derived foam cells^79^. Moreover, Ma et al. stated the downregulation effect of Liraglutide on BACE1 in HT-22 cells (immortalized hippocampal neuron cell line) exposed to high glucose, which came in accordance with our results^80^. To our knowledge, this is the first study to investigate the role of Liraglutide in modulating the LPA/LPAR1 pathway. Owing to the ubiquitous expression of GLP-1/GLP-1 receptors in the CNS, Liraglutide, a GLP-1 analog, has shown promise in preventing the development and/or progression of the AD-like phenotype in preclinical models. In the present study, Liraglutide therapy efficiently ameliorated AlCl_3_-induced AD in rats. Along with the suppression of hippocampal oxidative stress (increased GSH and decreased MDA levels) and enhancement of cerebral cholinergic transmission (decreased AChE activity), control of the oxLDL/LPA/LPAR1/BACE1 pathway appears to play at least a partial role in mediating the neuroprotective effects of Liraglutide against AlCl_3_-induced AD. These effects were validated through behavioral testing and histopathological examination

### Limitations of the study

Our choice for the AlCl₃-induced model was based on robust evidence of high reproducibility, ease of induction, short timeline to pathological and behavioral alterations, lower mortality rates, availability, and lower induction cost. The fact that Al is the most abundant neurotoxic metal, is strongly associated with AD pathogenesis, can cross the blood-brain barrier, and accumulate in the brain, all added to our model preference. Though we (and others) could recover pathological evidence of amyloid plaque-like depositions and NFTs in the AlCl₃-induced model; one limitation that was previously noted for this model is that the density and/or structure of these depositions might differ from those found in human patients and other transgenic AD models. As no experimental model can perfectly replicate all the neuropathologic features that occur in AD, our plans include validating the potential therapeutic effects of Liraglutide in different transgenic AD models. A future aspect of our research is also to establish experiments with a specific LPAR1 antagonist to confirm causality for LPAR1’s role to clarify whether Liraglutide’s effect on the oxLDL/LPA/LPAR1/BACE1 pathway is a direct or downstream consequence of its broader anti-inflammatory/anti-oxidative actions.

## Supplementary information

Reporting Summary

## Data Availability

The raw datasets generated and analyzed during the current study are available in the Figshare repository at [10.6084/m9.figshare.30008767]^81^. All other data supporting the findings of this study are available from the corresponding author upon reasonable request.
